# Night Work and Breast Cancer Risk in Nurses: Multifactorial Risk Analysis

**DOI:** 10.3390/cancers13061470

**Published:** 2021-03-23

**Authors:** Juan Gómez-Salgado, Javier Fagundo-Rivera, Mónica Ortega-Moreno, Regina Allande-Cussó, Diego Ayuso-Murillo, Carlos Ruiz-Frutos

**Affiliations:** 1 Department of Sociology, Social Work and Public Health, Faculty of Labour Sciences, University of Huelva, 21007 Huelva, Spain; frutos@uhu.es; 2 Safety and Health Postgraduate Programme, Universidad Espíritu Santo, Guayaquil 092301, Ecuador; 3 Health Sciences Doctorate School, University of Huelva, 21071 Huelva, Spain; javier.fagundo308@alu.uhu.es; 4 Centro Universitario de Enfermería Cruz Roja, University of Seville, 41009 Seville, Spain; 5 Escola Superior de Saúde, Universidade Atlântica, 2730-036 Barcarena, Portugal; 6 Department of Economy, Faculty of Labour Sciences, University of Huelva, 21007 Huelva, Spain; ortegamo@uhu.es; 7 Department of Nursing, University of Seville, 41009 Seville, Spain; rallande@us.es; 8 General Secretary, General Council of Nursing, 28023 Madrid, Spain; d.ayuso@consejogeneralenfermeria.org

**Keywords:** night work, shift work, nurses, breast cancer, risk factors for breast cancer, occupational health, occupational hazards, breast cancer prevention, circadian disorder

## Abstract

**Simple Summary:**

The incidence of breast cancer worldwide has increased in recent decades in women, and shift work, which implies night work, has been identified as a likely carcinogenic factor for humans due to the biological and lifestyle alterations it entails. Nurses, mainly represented by the female collective, undertake working conditions with intensive rotative and night shifts. Therefore, it is appropriate to describe the association between breast cancer and rotating night shifts in nurses, analysing the effect of consecutive night shifts, sleep disruption, work-family stress and medication intake, among others. This article demonstrates that preventive measures must be considered from healthcare managers to reduce occupational breast cancer hazards. In this way, it is important to consider the constant exposition of nurses to a stressing environment while stopping their biological clock to maintain the continuity of care “24/7”.

**Abstract:**

Night work has been highlighted by the International Agency for Research on Cancer (IARC) as a likely carcinogenic factor for humans, associated with breast cancer and professions that require continuity of work. Knowing the impact that short and long-term night work has on the nurses’ collective seems a priority, therefore, this study aims to analyse the relationship between night work and the development of breast cancer risk factors in nurses. For this, a cross-sectional study through an online questionnaire on breast cancer risk variables and working life was designed. The study was conducted in Spain and the sample consisted of 966 nurses, of whom 502 were healthy participants and 56 were breast cancer patients. These two groups were compared in the analyses. A descriptive analysis was performed, and the relationship was tested using χ2 independence test and OR calculation. The CHAID (Chi Square Automatic Interaction Detection) data mining method allowed for the creation of a segmentation tree for the main risk variables. The most significant risk variables related to working life have been the number of years worked, nights worked throughout life, and years working more than 3 nights per month. Exceeding 16 years of work has been significant for women and men. When the time worked is less than 16 years, the number of cases increases if there is a family history of cancer and if there have been more than 500 nights of work. High-intensity night work seems more harmful at an early age. The accumulation of years and nights worked increase the risk of breast cancer when factors such as sleep disturbance, physical stress, or family responsibilities come together.

## 1. Introduction

Breast cancer (BC) is the most common type of cancer among women and the second in incidence behind lung cancer. Approximately 2.1 million cases were reported worldwide in 2018 [1]. In Spain, BC is the leading cause of death from cancer among women [2]. In 2019, 32,536 new cases were diagnosed [3], which means a rate of 140 BC cases per 100,000 women in this country by 2020 [4]. The mean age of the onset is placed between 45–65 years [2,5], with an increase in incidence from the age of 75 onwards [2]. The current survival rate in Spain is 83% due to, among other factors, prevention campaigns, early diagnosis, and the latest therapeutic advances [6,7,8].

One of the most assessed risk factors for BC in recent years has been night work, which is defined as one that requires “at least three hours of work between midnight and 5 a.m.” [9,10,11] and is characterised by several elements: the duration of the work shift, the distribution of shifts (e.g., sequence morning-afternoon-night; nights only, etc.), the number of consecutive nights worked, the total number of nights worked per month and per year, the start and end time of the shift, the number and schedule of days off, and the regularity or irregularity of the staff rotating shift system [12,13]. Night work is common in professions that require continuity such as healthcare, industrial, transport, communications, leisure, and hospitality sectors, as their work performance requires continuity 24 h a day [13,14,15,16,17]. Nursing stands out among these working groups, as it is the most common profession that implies women working at night [18].

In 2010, and after undergoing a review in 2019, night work was classified by the International Agency for Research on Cancer (IARC) as a likely carcinogenic factor for humans (Group 2A), based on limited evidence of epidemiological studies and sufficient evidence from animal models [9,14,15]. In general, it was concluded that there is a positive association between night shift work and BC due to the biological and lifestyle alterations it entails, but studies to date and with the methodology used have not yet been able to isolate night work as a cancer-independent causal factor. Therefore, the presence of confounding factors should not yet be excluded, and further research is required [14,15,18,19,20,21].

These carcinogenic effects of shift work on the organism are thought to be related to a desynchronisation between the biological clock and the daily cycle of light-darkness due to exposure to artificial light [9,10,11,12,13,14,15] and sleep deprivation [22,23,24] during the night period. Such circadian alteration appears to be essential in influencing various levels of the body’s metabolism, among others affecting the nocturnal secretion of melatonin [13,25,26,27]. This hormone is produced in the pineal gland during the darkness period and is associated with the control of sleep-wake cycles (circadian rhythms) [26,28] and with anti-cancer properties due to its oestrogen-modulating action [25,29,30].

Consistent findings showed that nurses working on rotating night shifts had a very strong association with BC as compared to nurses who worked during the day or never did so at nights [19,31,32,33,34,35]. This relationship corresponds with the fact that 75–80% of BC cases identified among nurses are luminal [36,37,38,39], possibly caused by the increase in oestrogens recorded in night shift workers (both at home and in the workplace) [29,31,32,33,34,40,41,42,43,44,45,46,47,48] by using biomarkers such as cortisol, 6-sulfatexymelatonine (aMT6s), or 17-β-oestradiol to measure the extent of circadian disruption [13,26,30,41,42].

Being identified as a possible carcinogenic factor for humans, it is necessary to understand the impact of night work and short and long-term circadian disruption on the already known risk factors for BC. Therefore, the objective of this research is to analyse the relationship between shift work, especially night shift work, and the development of BC in nurses, as well as to study and classify those BC risk factors associated with this work organisation.

## 2. Materials and Methods

### 2.1. Design and Sample

Cross-sectional study through an online questionnaire. The target population was the professional group of Registered Nurses, both men and women, currently working in Spain, which amounts to 316,094 subjects according to data obtained from the Spanish Institute of Statistics in 2020 [49]. The sample selection was made by non-probabilistic snowball sampling, estimating the optimal size at 980 nurses with a 95% confidence level, 3.5% accuracy, and 20% adjustment for losses. The sample size estimation made contrasting healthy people with breast cancer cases possible, with a sufficient number of individuals per group.

The sample included Registered Nurses who were working in private and public centres in Spain, whether they performed night shifts or not, including those who re-joined after previous sick leaves or who had worked shifts in previous periods. Non-registered nurses and nursing undergraduate students were excluded.

### 2.2. Instrument

To develop the questionnaire for this study, the main risk variables were identified after a bibliographic review [21]. In this sense, the variables already validated were included as their authors recommend, and those variables for which there is no validated instrument were translated and adapted ad hoc, through internal validation. Subsequently, the final questionnaire was formed by 43 items distributed in seven sections: sociodemographic aspects, general data on cancer, lifestyle habits, family responsibilities, sleep and rest, consumption and exposure to tobacco (these questions were extracted from the Nebot et al. [50] questionnaire), and labour information. The validation of the final instrument was carried out by a panel of 10 experts made up of healthcare professionals and university professors linked to the areas of occupational health and public health. The experts participated in two rounds of discussion and reached consensus on the final items for the study (See File S1: Study questionnaire).

### 2.3. Variables

The following variables were considered: *sociodemographic data* (age, sex, marital status, and level of studies), *general data on diseases and cancer* (current disease, oncological disease, mammograms, oral contraceptives, first-degree familial cancer, work exposure to electromagnetic and/or cytostatic fields), *lifestyle habits* (BMI, physical activity at work and during free time), *family burdens* (children under the age of 14, and care for dependents at home), *sleep patterns* (regular rest time, mean number of hours of night sleep, intake of sleep medication), *exposure to tobacco* (consumption habits, exposure to tobacco in the workplace and at home), and *labour information* (type of entity—primary or specialised care, public or private; position in the nursing area; service/area/unit of work; time worked in the current company; type of schedule and/or shifts; number of accumulated years of work (throughout life); number of years working regularly more than 3 nights per month; number of worked nights accumulated throughout life; age of initiating night shifts; and sick leaves throughout life and in the last year).

These variables were identified as effect modulators in the analyses: sick leaves (number of leaves and number of days off), taking sleep medication, and number of mammograms.

### 2.4. Procedure of Data Collection

The study development took place from December 2019 to November 2020. Google Forms© (Google, Mountain View, CA, USA) was used to create the online questionnaire. Participants could not access the questionnaire until they had previously done the following: (a) Having read and understood an introductory letter to the study and its objectives; (b) Having confirmed voluntary and anonymous participation in the study; (c) Declaring working as a nurse in Spain and being currently registered. The data obtained from the completion of the questionnaire was automatically entered on an individual and anonymous database in Excel (Microsoft, Redmond, WA, USA). Once the number of expected results was obtained, the Excel sheet was dumped to the SPSS (IBM, Armonk, NY, USA) database for its statistical analysis.

The online questionnaire was provided via email to registered nurses in the Spanish General Nursing Council and through the social networks of official entities and professional groups and of renowned prestige in the area of nursing in Spain.

### 2.5. Statistical Analysis

The descriptive analysis of all the variables was performed by determining absolute frequencies and percentages of the above-mentioned variables. To contrast the relationships between the variables and BC, the χ2 independence test was used, determining estimated risks from the Odds Ratios (OR) and their confidence intervals. All analyses were performed separately for women and men. In order to delve into the “age” variable, a further bivariate analysis of the significant risk variables categorized by age was performed, thus allowing to display age-adjusted OR. The analyses were carried out through the SPSS 26.0 statistical software (IBM, Armonk, NY, USA) and R Commander [51].

A hierarchical classification technique was executed after the descriptive analysis, using the CHAID (Chi Square Automatic Interaction Detection) data mining method. The algorithm determined which risk variables played a significant role in BC, using the chi-squared independence test, and choosing the most significant factor(s) with the smallest *p*-values (lower or equal to the significance level set at α = 0.05). The sample is divided according to the levels stablished by the chosen factors, and each resulting group repeats this division until it is not possible to continue dividing further or no other significant factor is found. This method did not require any restrictive assumptions (such as variable or residual normality) and permitted to create a classification tree which would be useful for guiding the causes and designing preventive actions towards BC.

### 2.6. Ethical Considerations

For this study, the Declaration of Helsinki 2004 was taken into consideration and explicit written permission was obtained from participants through their informed consent for the confidential use and processing of their data in accordance with the Organic Law on Protection of Personal Data and the Guarantee of Digital Rights. Data are guaranteed to be duly guarded by the research team. Ethical approval was obtained from the Research Ethics Committee of the Spanish General Nursing Council, as well as from the Research Ethics Committee of the province of Huelva, belonging to the Regional Government of Andalusia (Spain) with code TD-CMTE-2020.

## 3. Results

### 3.1. Sociodemographic Data

The questionnaire was answered by a total of 966 nursing professionals aged 41.21 (SD = 10.60), of whom 10.35% were male and 89.65% were female. Of the responses, 51.97% were from healthy individuals (those who had never had cancer or any other type of disease), 10.25% from those who had or ever had some type of cancer, and 37.78% from participants with another type of disease. Of those who had or had had cancer, 56.57% corresponded to BC (Table 1).

### 3.2. Descriptive and Comparative Analysis between Healthy People and People Who Had or Ever Had Breast Cancer

Healthy individuals (502 responses) and BC patients (56) were compared two-dimensionally with the main variables of interest for the study (Table 2).

Of the nurses, 10.39% were men and five cases (8.93%) of male BC were detected; there were no significant differences (*p* = 0.705) by sex between healthy nurses and those affected by BC. In relation to the variable “age”, the main analysis did not reveal significative differences (*p* = 0.367) between the age groups created by dividing according to the median (41 years), as seen in Table 2. Subsequent bivariate analysis categorized by age revealed only marital status (χ2 = 58.212; *p* < 0.001; OR = 0.257, 95%CI = 0.180, 0.367) and intake of sleeping medication (χ2 = 6.711; *p* = 0.010; OR = 1.728, 95%CI = 1.139, 2.620) as significant in relation to BC. Relevant differences between healthy participants and BC patients were also detected in those with a partner (*p* = 0.041), OR = 1.848, 95% CI = (1.018, 3.355), but not according to the academic degree (*p* = 0.653) (Table 2).

Having had a mammogram is presented as a modulating variable in cases of BC (*p* < 0.001). The mean number of mammograms in the sample was 2.27 (SD = 4.43), although 57.55% of the sample had never performed a mammogram and this number increased to 9 (SD = 6.83) in participants with BC. Familial BC also showed statistical significance (*p* = 0.005) and increased risk, OR = 2.511, 95% CI = (1.293, 4.879). However, no significant differences were found regarding the use of oral contraceptives or regular exposure to electromagnetic or cytostatic fields (Table 2).

In terms of lifestyle habits, body mass index (BMI) showed significant differences (*p* = 0.045). The higher number of BC cases was found among normal weight individuals, followed by cases with overweight and obesity. Statistically significant differences (*p* < 0.001) were also detected depending on the type of physical activity at work, the percentage of cancer cases being higher in those considering the activity to be “very hard”. The mean number of hours of physical exercise the week before the questionnaire was 3.07 h (SD = 3.80); no significant differences were found for more than 2 h (median) of physical activity or less (*p* = 0.631) (Table 2).

With regard to family burdens, 40.3% of participants had children under the age of 14 and 10.4% cared for dependents at home (elderly or family members with a serious illness and disabilities). Based on the results, having children under the age of 14 did not report a statistically significant association with BC (*p* = 0.684). Nevertheless, caring for dependents was relevant in this association (*p* < 0.001), with an OR = 3.470, 95% CI (1.759, 6.844). More specifically, 24.1% of professionals who had or ever had BC cared for dependents, as compared to 8.4% who had or ever had BC and did not have dependents to care for.

In relation to sleep habits, the mean hours of rest amounted to 6.30 (SD = 1.09), with 43.2% of nurses considered to have a regular sleep schedule and 79.2% who claimed not to take any sleep medication. In studying the association of these variables with BC cases, taking sleep medication also proved to be a modulating variable (*p* < 0.001), OR = 7.243, 95% CI = (4.047, 12.964) (Table 2). However, there were no significant differences with a regular sleep schedule (*p* = 0.278) (Table 2).

With regard to tobacco exposure and consumption, the mean number of hours shared with smokers was 4.40 (SD = 4.63), with a mean of 0.27 h of exposition in the workplace (SD = 0.91). Of the respondents, 53.9% said they have never smoked; at the time of the questionnaire, 14.7% claimed to smoke every day and 4.3% did it occasionally, not associating these data with having or ever had BC (*p* = 0.953 and *p*= 0.841, respectively). Statistically significant differences were found regarding complying with the smoking ban in the workplace (*p* = 0.010) and with the frequency of exposure to tobacco smoke at home (*p* = 0.001) (Table 2).

With respect to data on the current work, the type of organisation (public or private), the level of healthcare attention, or whether working full-time or part-time did not have an association with having or having had BC. Nonetheless, the time worked in the current company (categorised according to the median time) and presented statistically significant differences (*p* < 0.001) when it was over 10 years. The responses to the current job schedule indicated that 82% of healthy nurses, compared to the 59% with BC, worked shifts (*p* < 0.001). Of healthy people, 83% were engaged in rotating shifts (*p* = 0.002), compared to the 66% with BC, and 70% of healthy participants performed night shifts (*p* < 0.001), compared to the 46% with BC (Table 2).

Considering the working history of the participating subjects, the total number of years worked is presented as the most significant variable in this category (*p* < 0.001). The mean number of years worked by the sample was 15.98 (SD = 9.6), being longer than 16 years (medium value) in 47.2% of individuals. Statistically significant differences were detected in relation to the number of years worked in those who had or ever had BC (mean = 26.1; SD = 8.1), and healthy respondents (mean = 15.0 years worked; SD = 9.2).

The percentage of cases with BC was also higher in professionals with 500 or more nights worked (*p* < 0.001) and in those who had been regularly working 3 or more nights per month for more than 10 years (*p* < 0.001). Throughout their working lives, 25% of nurses had worked up to 158 nights, 50% had done at least 500 nights, and 25% of them had worked 1000 or more nights. The mean number of nights worked was 663.4 (SD = 668.5); in the case of healthy subjects, it was 627.9 (SD = 639.4), and 1017.4 nights in those who had or ever had BC (SD = 837.9), showing a statistically significant difference (*p* < 0.001). The risk of BC was higher in those who had been working for more than 16 years, 3 or more nights per month for more than 10 years, and more than 500 nights. Of the respondents, 2.3% claimed not to have worked night shifts. Among those who had done so, 75% began working night shifts at the age of 25 or younger (median). There were no significant differences (*p* = 0.919) when considering the age of the onset of night work.

The cumulative number of sick leaves and the number of days off, both in the last year and throughout their professional life, was related to having or having had BC (*p* < 0.001 in all cases). Most BC cases accumulate more than two sick leaves (19.7%) and more than 40 days of leave (18%). In the last year, the number of sick leaves and days off increased among those with BC, as compared to the healthy group (Table 2).

### 3.3. Segmentation Tree Based on Risk Factors

**Figure 1:**
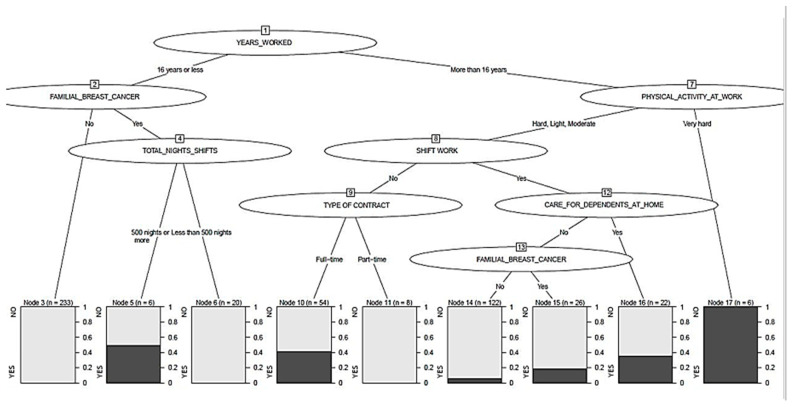
Segmentation tree of breast cancer and work-related factors.

Only the most relevant BC risk variables have been considered for the development of the segmentation tree (Figure 1). Sixty-one individuals were excluded from the sample, as some of the questions related to risk factors were not answered.

The number of years worked is displayed on a first node of the segmentation tree as the most significant variable. For 52.1% of individuals with 16 years of work or less, BC cases were mediated by familial BC. The percentage of BC was 0.9% if there was not familial BC cancer and, segmented by the total worked night shifts, the percentage of cases was 50% when nurses with family history of BC had worked 500 nights or more. No case appeared when less than 500 nights had been worked.

When the time worked exceeded 16 years, BC cases were mediated by physical activity at work. One hundred percent of those who considered their physical activity at work to be “very hard” had BC, while for those whose physical activity was perceived as light, moderate, or even hard, cases of BC were mediated by performing shift work at the time of the study. Among those who did not perform shift work but worked part-time, there were no cases of BC, and the percentage of cases increased to 42.6% in those who worked full-time. In the case of performing shift work, the percentage of BC cases was 36.4% in those who care for dependents but decreased to 19.2% in those who did not care for dependents although having a family history of BC. If they did not have a family history of BC, even if shift-working, cases came down to 6.6%.

### 3.4. Sex Segmentation Analysis

Sex segmentation allows to assert that the descriptive, two-dimensional, and segmentation analysis for women show similar results to the overall group (women account for 89.6% of cases under study). In the case of men, an association was detected between having BC and academic degree (3.862; *p* = 0.049), mammography (Fisher’s test *p* < 0.001), sleep medication (48.462; *p* = 0.006), years worked (4.766, *p* = 0.029), and variables related to sick leaves. The association with working nights shifts at a significance level of 7% (3.365, *p* = 0.067) could be confirmed.

**Figure 2:**
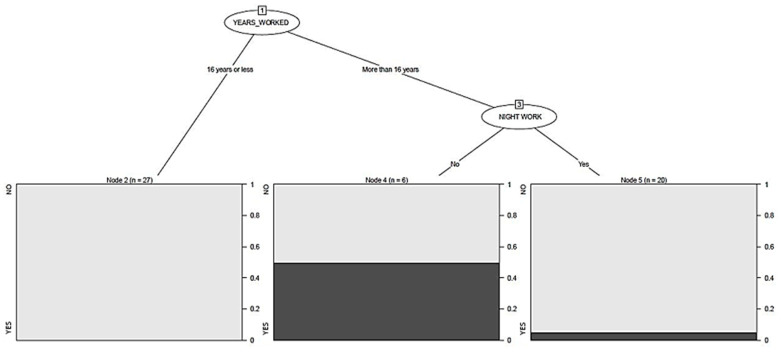
Segmentation tree for male breast cancer.

The number of years worked was presented as the main segmentation variable among men. There were no cases in those who had worked for 16 years or less. Performing night work at the time of the study proved to be a mediating variable for those who worked over 16 years. Fifty percent of the male nurses who did not work at night had BC, as compared to 5% of cases who did work at night (Figure 2).

## 4. Discussion

The risk of BC for people with a first-degree familial BC ranges from 50–80% over lifetime, as some authors note [27,52]. Similarly, the wider the family history of BC, the greater the risk, depending essentially on age at the diagnosis, the number of affected family members, and the generational distance from those affected [47,53,54]. The results of this study indicate that a first-degree familial BC increases the risk of developing BC (OR = 2.511), which would be a consistent result with the available evidence, albeit with a weaker association.

This relationship to familial BC would also apply to male BC, which resulted in five cases in this study (8.92% of the BC cases tested); even if it only accounts for 1% of all male cancers worldwide [55,56]. Longitudinal studies indicate that its incidence is growing similarly to female BC and that the survival rate does not differ between sexes [57,58]. Genetic predisposition, aging, first-degree family history, and radiation exposure are the main risk factors for BC among men [59,60]. Specifically, the BRCA-1 and BRCA-2 genes are related to most cases and are associated with a younger age in diagnosis, with positive hormone receptors of oestrogen and progesterone (ER+ and PR+), and with negative HER2 (human epidermal growth receptor factor 2). Male BC has also been associated with factors that can increase oestrogen levels such as taking hormone medications, being overweight, consuming large amounts of alcohol, or suffering from liver disease [61,62]. The present study, as shown in Figure 2, notes that working 16 years or more was statistically significant for the risk among male nurses, which is mainly explained by the age of the subjects, as mentioned earlier [59,60]. On the other hand, 5% of cases of male BC worked night shifts and 50% of cases did not work nights, similar to what happened in the general sample.

In addition to sex, as various studies indicate, the risk of developing BC increases with age, especially from the age of 50, when there is the highest incidence in women [5,63,64,65]. However, the growing number of BC cases occurring in young women is remarkable [5,65] and certain factors are closely associated, such as adolescent alcohol consumption [66], breast cancer subtype (mainly ER−, PR− and HER2+) [67,68] or the presence of genetic alterations [65,69]. In this sense, our study revealed no differences according to age between healthy participants and BC patients (*p* = 0.367). Nevertheless, due to the relevance of the age factor on the literature, it was considered appropriate to perform a further bivariate analysis categorized by age of the significant risk variables in Table 2 for healthy and BC cases. This analysis concluded that only marital status (χ2 = 58.212; *p* < 0.001; OR = 0.257, 95%CI = 0.180, 0.367) and sleep medication (χ2 = 6.711; *p* = 0.010; OR = 1.728, 95%CI = 1.139, 2.620) showed significant differences adjusting by age in relationship with BC.

According to data from the Danish Nurse Cohort, female nurses working night shifts had higher all-cause mortality than those working day shifts [70]. In this same line, the so-called shift-work disorder that occurs as a result of workers’ circadian disruption [12,13,71] not only implies altered sleep patterns, insomnia, frequent snoring, excessive daytime sleepiness, and a higher prevalence of depressive symptoms [17,71,72], but also involves decreased physical activity [11,73,74] and poorer dietary control [11,16,20,24,74,75], with increase of the risk of cardiovascular disease (CVD) and diabetes [70,76]. Despite this, no association was found in other studies between shift work and all-cancer mortality [70,77]. In the present study, a higher number of cases were identified in people with normal or elevated BMI, and physical activity in the working context was statistically significant in BC cases when it was classified as “very hard” (Figure 1). Regarding this, several studies agree on the benefit of having an active physical activity, both occupational and in leisure time, to reduce the risk of BC [78,79]. However, it is necessary to regulate the intensity of the leisure-time physical activity since work-related overload and exertion is associated with health problems, such as increased risk for CVD [80], and with insufficient rest after a tough shift work, which is associated with an increased risk of insomnia [81]. Therefore, intense occupational physical activity will require gentle physical activity during free time, while low or sedentary occupational activity is ought to be balanced with moderate recreative physical exercise. All accompanied by a sufficient rest time appropriate to the effort made [81].

Insomnia problems are approximately three times higher among cancer patients than in the general population [23,82,83], and impaired sleep patterns persist in more than 50% of BC survivors due to the multiorgan component of the disease, the deterioration of the immune system, or the alteration in melatonin release, among other factors [13,25,26,27,82,84,85,86,87,88]. In this way, it has been suggested that the relationship between night work and sleep rhythm disruption in nurses implies an increased risk of BC [17,89] and could also involve exposure to other risk factors such as stress, self-medication, tobacco abuse, or the use of psychoactive substances [23].

In this sense, 56.8% of the study subjects claimed to have an irregular rest schedule, although this was not significant for BC cases, and only 20% of respondents resorted to sleep medication, whereas this variable was more significant for those who had or ever had BC (*p* < 0.001; OR = 7.243) also in relationship with age over 41 years (OR adjusted by age = 1.728, 95% CI = 1.139, 2.620). Thus, it is noted the relationship with other studies that associate hypnotics with cancer cases due to insomnia and impaired sleep patterns that occur during any stage of the disease and that persist in survivors [82,84,85,88]. Other studies have recommended the use of melatonin as a supplement to try to adjust sleep time [90] or the use of stimulants such as caffeine to reduce sleepiness, although there is no robust evidence at this moment [91,92].

As for smoking, the results of recent studies are consistent with the increased risk of BC in ER+ and PR+ tumours that occur in active and passive smokers, as well as in those who stopped smoking up to 20 years ago and in women who smoked between menarche and the first full-term pregnancy [27,93,94]. In the present study, smoking or having smoked showed no significance, although the risk of BC has been linked to passive exposure to tobacco both in the workplace and at home.

On the other hand, according to the study data, having children (under the age of 14) does not seem statistically significant in reducing the risk of BC (*p* = 0.684; OR = 1.123). These results appear to contradict established risk factors for hormone-related BC such as nulliparity [47] or giving birth at late age [27,47,95]. Other studies found that the risk of developing BC was higher among women who had worked night shifts for more than 4 years before their first full-term pregnancy, a period in which the body growth may not yet be completed and which makes the person more susceptible to circadian disruption [19,70,96]. Following this, the exposure to oestrogen-progestogen contraceptives and hormone replacement therapy, related to a risk in hormone and non-hormone receptors [97], have not been statistically significant in this study. However, this type of treatment has been used very little in Spain [98,99].

The association with the working life, in this study, highlights that more than 95% of participants had worked shifts and nights at some point in their careers, especially at early ages, as indicated in other studies [70]. Nevertheless, the percentage of nurses who were working shifts, nights, and rotating shifts at the time of the questionnaire was smaller in the BC cases group than in the group of healthy nurses. This situation may have been possible because those nurses with BC could have received a modification or compensation on behalf of their workplace organisation when they were diagnosed or re-joined after the sick leave, exempting them from working rotating shifts and night shifts in order to create a less aggressive work environment for the worker, as described before [42].

The working history is of great interest in this research. The number of years worked, the number of nights worked over life, and the number of years working more than 3 nights per month are the main statistically significant occupational variables for BC cases in this study. Among them, the time worked is presented as the most significant variable, with the number of nights worked in the first 16 years of professional career playing a remarkable role. In this way, relationships were found when nurses worked less than 16 years but there was a family history of BC and more than 500 nights were worked, indicating high exposure to night shifts during the first years of the working history of a person with certain risk (Figure 1).

Several authors [31,32,33,34,100] confirm the risk of BC among nurses working on rotating night shifts at least 3 nights per month for 20 years or more, particularly those who started in their young adulthood (before the age of 30). On the other hand, those characteristics of night work that are indicative of high intensity of exposure (3 or more nights per week), long duration of night work over life (at least 10 years in a row), and long night shifts (10 or more hours) were associated, to a greater or lesser extent, with an increased risk of BC in premenopausal women at 5 years of their working life [17,19,31,45,92]. In contrast, recent studies [18,70] showed no association between BC, night shifts, and cumulative shifts for 20 years or more.

Regarding the workplace, no differences were found between private and public centres or between working in a hospital, primary care, emergency department, or any other specialized sector. Specifically, exposure to electromagnetic fields or cytostatic medication was not significant in this study (*p* = 0.944 and *p* = 0.916, respectively). However, a recent study [101] discussed how melatonin, cortisol, and other serum markers were altered in radiation specialist nurses and night-shift nurses. Plasma levels of melatonin were observed to be higher in radiation specialist nurses (suggesting a mechanism of adaptation to oxidative stress induced by low doses of radiation), and significantly lower in night-shift nurses as compared to those of the day shift, which probably reflects a circadian alteration. Likewise, night work was related to high levels of cortisol.

The results of the present study have identified that people who have had BC often have long-term sick leaves and a large number of leaves interspersed with working periods. These results are in line with those proposed by López-Guillén and Vicente in a literature review of returning to work following a BC process [102]. Similarly, they conclude that these pathological processes cause a significant number of sick leaves in Spain, so the reasons for this incidence in sick leaves should be studied in depth, understanding that they may arise from emotional issues, adaptation to the new body image, or due to the adverse effects of the chemotherapy treatment.

Finally, it may be suggested that intense physical activity at work and prolonged working history, for more than 16 years, increase the risk of BC among nurses (Figure 1). Besides, other variables are associated such as nights worked, stable work (full-time), couple relationship, and care for dependents at home, perhaps indicating that from a mature age, new stressing and time-consuming factors come together, which could lead to work-family conflicts [103]. As happens in other similar cultures [104], Spanish families maintain a close relationship with all their members, and it is common for women to take responsibility for caring older family members at home rather than resorting to Retirement or Elderly Care Centres. This difficult work–life balance can affect both the role of nurses [105] and family stability [106], as well as assuming lack of time for leisure and self-care [107], tiredness, and sleep disorders [108].

### 4.1. Limitations

In order to analyze the results of this study without falling into interpretation biases, it should be kept in mind that this is a cross-sectional study and that the responses obtained in the questionnaire refer to data on current work and on the full career experience. Due to the methodology used for this research, the effect modification has not been assessed, no control has been kept on the study variables, nor is there a control group to definitively conclude the association between night shifts and the incidence of BC. Similarly, the genetic variable and disruption of circadian clock gene regulation, which have shown to have an impact on the incidence of cancer processes, have not been experimentally analyzed in this study and there was no control of predisposing genetic mutations, or blood and biopsy anomalies. In addition, the breast cancer diagnosis was not clinically confirmed because data was collected using self-reported information.

On the other hand, the dissemination of the questionnaire should have been more comprehensive as it could potentially represent participation bias in this study. In this way, participation may have been weaker among those nurses who were sick or on sick leave during the data collection period since, perhaps, they refused to respond because of their health situation. However, the sample and number of responses has been estimated as sufficient to overcome this issue.

It is also important to consider the recall bias as another study limitation related to the retrospective study design. In this sense, some variables such as BMI or free-time physical activity could have been influenced by certain factors regarding the BC cases, for instance, chemotherapy treatment.

It is known that male BC has a low global incidence and is diagnosed mainly after the first clinical manifestations, as screening is not routinely performed and breast imaging plays a limited role, even when its usefulness is demonstrated [61]. From the outset, the present research considered the possibility of cases appearing in male nurses and, therefore, a methodology of sampling and analysis of the main inclusive risk factors for both sexes was carried out. The five cases of BC identified in men have been a relevant finding of this research, and main associations with variables such as academic degree, mammograms, sleep medication, years worked, and sick leaves have been found, resulting in the number of years worked as the most significant factor. It should therefore be noted as a limitation that the number of cases in men has been insufficient to establish generalized conclusions and that, on the other hand, no other highly related variables such as epigenetic alterations or tumor characteristics have been analyzed.

### 4.2. Implications for the Practice and Future Perspectives

At the organisational level, certain preventive measures could reduce the possible negative effects of shift work:

- Improved shift work schedule, rotating periods, and breaks: work schedules should be adapted to allow balance between personal life and the adjustment of circadian rhythms before the rotating schedule goes to the next pattern. The shifts are recommended to be adjusted forwards (morning, afternoon, night) and it is also recommended to have a rest period of 24 h after each night shift, increasing the rest time as more consecutive nights have been worked. Ten hours, 12 h, or 24 h-shifts allow fewer consecutive shifts and longer rest periods, although it can cause fatigue due to the high number of working hours.
- Improved facilities: for example, providing adequate lighting, temperature, and ventilation. If possible, offering facilities that allow nurses to rest. The cafeteria or catering for workers should be provided with healthy products.
- Improving the relationship between workers and the company management would be important to increase job satisfaction and compliance with shifts, breaks, and the rotating schedule.

This study has led to a descriptive image that shows that shift work is a reality for a large percentage of nurses. Future research needs to strengthen methods that highlight the clinical consequences of shift work and night work. Besides, the genetic markers of BC, biomarkers of circadian hormonal disruption, and tumour characteristics of all cases should be considered. On the other hand, assessing the interactions between the already known BC risk factors through a multivariable modelling approach and establishing risk assessment models related to shift work should remain a priority.

Moreover, this study has considered the presence of BC also in men through an inclusive methodology for both sexes. With this, a surprising number of positive cases has been discovered in men, which probably would have gone unnoticed in a woman-focused research. These cases have been analysed and suggest the need to continue researching on BC in male nurses to assess whether there exists an influence of shift work on the same variables as in female nurses.

## 5. Conclusions

The results from this descriptive study suggested that night work should be considered as a risk factor for BC development among nursing professionals, consistent with previous health studies on this group of workers. However, this study alone cannot definitely conclude these outcomes because of the cross-sectional nature of the study design. Certain variables are associated with this fact: Having a first-degree family history of cancer, having dependents to care for, BMI, level of physical activity, exposure to tobacco, shift work, rotating shift work, years worked, nights worked over life, and years working and doing more than 3 nights per month. In addition, the presence of a family history of cancer has proven to increase the incidence of BC in both female and male nurses and has reinforced the idea that night work involves an increased risk of other health problems, as compared to daytime work. The consumption of sleep medication, mammograms, and taking days off are presented as modulating variables of the incidence of cancer among nursing professionals.

Currently, healthy nurses in this study work a greater number of shifts, nights, and rotating shifts than those who have or ever had BC, so the effects of shift work become apparent when the working career of these professionals is checked. The risk of BC is highlighted when more than 500 nights, over than 16 years, or more than 3 nights per month for more than 10 years have been worked. However, the effects of intensive work shifts and the excess of consecutive nights could be especially significant among young nurses, especially in those with a family history of BC who began to work nights before the age of 22.

The current nursing profession distributes its work throughout 24 h a day to ensure continuity and the highest quality of care; it would therefore be beneficial to implement preventive measures that minimize the effects of shift-work alterations to reduce the incidence of BC among nurses.

## Figures and Tables

**Table 1:** General descriptive analysis (N = 966).

Health Status	Number of Cases	Percentage	Age (Mean)	Age (SD)
Healthy	502	51.97%	41.29	10.66
Cancer	99	10.25%	41.89	10.06
Breast cancer	56	5.80%	41.41	10.63
Male	5	0.52%	38.40	11.46
Female	51	5.28%	41.71	10.50
Other cancer	43	4.45%	42.51	9.23
Other illness	365	37.78%	40.91	10.64
Overall	966	100.00%	41.21	10.60

**Table 2:** Bivariate analysis of risk variables between healthy nurses and nurses with breast cancer (N = 558).

	N (%)	Breast Cancer (%) (N = 56)	Healthy (%) (N = 502)	χ2	*p*	Odds Ratio (CI = 95%)
Sex						
Male	58 (10.4)	8.6	91.4	0.144	0.705	0.831 (0.318, 2.173)
Female	500 (89.6)	10.2	89.8			
Age						
41 years or younger	281 (50.4)	8.9	91.1	0.813	0.367	0.775 (0.445, 1.350)
Older than 41	277 (49.6)	11.2	88.8			
Marital status						
With partner	317 (56.8)	12.3	87.7	4.178	0.041	1.848 (1.018, 3.355)
Single	241 (43.2)	7.1	92.9			
Academic degree						
Nursing degree	303 (54.3)	10.6	89.4	0.203	0.653	1.137 (0.651, 1.985)
Nursing speciality, Master or Doctorate	255 (45.7)	9.4	90.6			
Hormonal-based oral contraceptives *						
Yes	334 (66.3)	11.7	88.3	0.594	0.441	1.272 (0.689, 2.350)
Never	170 (33.7)	9.4	90.6			
Mammography *						
Yes	211 (42.5)	26.1	73.9	**	<0.001	0.739 (0.682, 0.801)
Never	286 (57.5)	0	100			
Familial breast cancer *						
Yes	72 (13.1)	19.4	80.6	7.814	0.005	2.511 (1.293, 4.879)
No	479 (86.9)	8.8	91.2			
Regular exposure to electromagnetic fields						
Ever	480 (86.0)	90.0	10.0	0.005	0.944	1.029 (0.467, 2.266)
Never	78 (14.0)	89.7	10.3			
Regular exposure to cytostatic medication						
Ever	392 (70.3)	90.1	9.9	0.011	0.916	1.033 (0.566, 1.883)
Never	166 (29.7)	89.8	10.2			
BMI						
Underweight	10 (1.8)	20.0	80.0	8.074	0.045	-
Normal	376 (67.4)	7.7	92.3			
Overweight	128 (22.9)	13.3	86.7			
Obese	44 (7.9)	18.2	81.8			
Physical activity at work						
Light	124 (22.2)	8.1	91.9	30.175	<0.001	-
Moderate	313 (56.1)	9.6	90.4			
Hard	113 (20.3)	8.8	91.2			
Very hard	8 (1.4)	75.0	25.0			
Physical activity last week (hours)						
Two hours or less	286 (51.25)	9.4	90.6	0.230	0.631	0.874 (0.530, 1.518)
More than 2 h	272 (28.75)	10.7	89.3			
Children younger than 14						
Yes	225 (40.3)	10.7	89.3	0.166	0.684	1.123 (0.642, 1.963)
No	333 (59.7)	9.6	90.4			
Care for dependents at home						
Yes	58 (10.4)	24.1	75.9	14.257	<0.001	3.470 (1.759, 6.844)
No	500 (89.6)	8.4	91.6			
Regular sleep schedule or pattern						
Yes	241 (43.2)	11.6	88.4	1.177	0.278	1.357 (0.781, 2.359)
No	317 (56.8)	8.8	91.2			
Sleep medication						
Yes	116 (20.8)	28.4	71.6	54.988	<0.001	7.243 (4.047, 12.964)
No	442 (79.2)	5.2	94.8			
Did you ever smoke?						
Yes	301 (53.9)	10.0	90.0	0.003	0.953	0.984 (0.565, 1.711)
No	257 (46.1)	10.1	89.9			
Currently smoking cigarettes						
Yes, everyday	82 (14.7)	8.5	91.5	0.347	0.841	-
Yes, some days	24 (4.3)	8.3	91.7			
No, I do not smoke	452 (81.0)	10.4	89.6			
The workplace complies with the smoking ban						
Totally	124 (22.2)	16.1	83.9	11.377	0.010	-
Almost always	239 (42.8)	10.9	89.1			
Hardly ever	141 (25.3)	6.4	93.6			
Never	54 (9.7)	1.9	98.1			
Exposition to tobacco smoke at home						
More than 5 h a day	22 (3.9)	31.8	68.2	15.967	0.001	-
Between 1 and 5 h a day	36 (6.5)	0	100			
Less than 1 h a day	42 (7.5)	7.1	92.9			
Never or hardly ever	458 (82.1)	10.0	90.0			
Organization *						
Public system	476 (85.9)	9.9	90.1	0.204	0.651	0.840 (0.394, 1.791)
Private system/Consortium	78 (14.1)	11.5	88.5			
Healthcare level						
Primary care	102 (18.3)	13.7	86.3	3.950	0.139	-
Hospital or Emergencies	435 (77.9)	9.7	90.3			
Other ***	21 (3.8)	0	100			
Years of experience in the current company						
10 years or less	270 (48.4)	3.0	97.0	28.985	<0.001	0.153 (0.071,0.329)
More than 10 years	288 (51.6)	16.7	83.3			
Type of contract						
Full-time	485 (86.9)	10.9	89.1	3.267	0.071	2.863 (0.871, 9.412)
Part-time	73 (13.1)	4.1	95.9			
Shift work at this moment						
No	114 (20.4)	20.2	79.8	16.315	<0.001	3.148 (1.765, 5.615)
Yes	444 (79.6)	7.4	92.6			
Rotating shift work at this moment						
No	104 (18.6)	18.3	81.7	9.597	0.002	2.519 (1.382, 4.592)
Yes	454 (81.4)	8.1	91.9			
Total years worked *						
16 years or less	280 (52.8)	1.8	98.2	36.842	<0.001	0.090 (0.035, 0.232)
More than 16 years	250 (47.2)	16.8	83.2			
Total years working regularly more than 3 nights per month						
10 years or less	317 (56.8)	4.7	95.3	22.870	<0.001	0.242 (0.131, 0.449)
More than 10 years	241 (43.2)	17.0	83.0			
Night work at this moment						
No	180 (32.3)	16.7	83.3	12.940	<0.001	2.708 (1.548, 4.735)
Yes	378 (67.7)	6.9	93.1			
Total night shifts *						
Up to 500 night shifts	302 (56.2)	5.3	94.7	12.187	<0.001	0.342 (0.184, 0.639)
From 500 night shifts onwards	235 (43.8)	14.0	86.0			
Age of first night shift						
22 or younger	289 (51.8)	9.7	90.3	0.080	0.777	0.923 (0.532, 1.604)
Older than 22	269 (48.2)	10.4	89.6			
Sick leaves *						
2 or less	342 (62.2)	3.8	96.2	36.977	<0.001	0.161 (0.084, 0.309)
More than 2	208 (37.8)	19.7	80.3			
Number of days on sick leave *						
40 days or less	284 (51.6)	1.1	98.9	47.121	<0.001	0.048 (0.015, 0.158)
More than 40 days	266 (48.4)	18.0	82.0			
Sick leaves in the last year *						
Without sick leave	385 (69.5)	4.4	95.6	40.782	<0.001	0.165 (0.090, 0.303)
With sick leave	169 (30.5)	21.9	78.1			
Number of days on sick leave in the last year *						
Never	379 (68.5)	4.5	95.5	36.134	<0.001	0.180 (0.098, 0.331)
Some day	174 (31.5)	20.7	79.3			

* The total number of cases does not correspond because this information is not collected in all subjects. ** Fisher. *** Other: teaching, management, business nursing and occupational health specialists. Note: BMI: Body Mass Index. <18.5 Underweight; [18.5,25) Normal; [25,29.9) Overweight; ≥30 Obese.

## Data Availability

All generated data is presented within this paper and its Supplementary Material. The research team and the University of Huelva are responsible of keeping the datasets of the study, which would be available for investigators under reasonable query.

## Supplementary Materials

The following are available online at https://www.mdpi.com/2072-6694/13/6/1470/s1, File S1: Study questionnaire.
