# Supplementary material for: Night Work and Breast Cancer Risk in Nurses: Multifactorial Risk Analysis
Source: Cancers (Basel). 2021 Mar 23;13(6):1470. doi: 10.3390/cancers13061470 (PMC8004617; doi:10.3390/cancers13061470)
Supplement: Supplementary file 1 [file cancers-13-01470-s001.pdf]

Study on breast cancer and shift-work in nurses

Dear Colleague,

The International Agency for Research on Cancer (IARC), in 2010, clasified shift-work as a likely carcinogenic factor for humans (Group 2A) due to the high incidence of breast cancer in women who work in night-shifts, such as nurses. Therefore, the objective of this project is to investigate the possible relationship between shift work, especially night work, and the development of breast cancer in this population.

The research team guarantees that all your answers will be treated anonymously and confidentially, being your participation totally voluntary.

To express your acceptance to participate in this study, please check the following box with a cross:

I agree to participate voluntarily in this study, once I received the information * ☐ Yes

PERSONAL DATA

Gender: ☐ Woman ☐ Man Age: ____ Years

Current marital status:

☐ Single ☐ Married ☐ Divorced ☐ Widow/er ☐ Other: ____________

Indicate the studies you have pursued and finished: *You can check more than one option*

☐ Graduate or similar ☐ University Master

☐ Nurse Specialist ☐ Doctorate

GENERAL DATA

Are you diagnosed with any illnesses? Indicate it: _________________

Have you had any cancers throughout your life? ☐ Yes ☐ No

Indicate type: ___________________

Has your mother or sister ever had breast cancer throughout their life?

☐ Yes, both ☐ Yes, my sister ☐ Yes, my mother ☐ No

Have you used oral contraceptives throughout your life? ☐ Yes ☐ No ☐ Not indicated

If you indicated «**YES**», for how long? ________ years.

How many mammograms have you done throughout your life? ______ mammograms.

In your work, have you had regular exposure to electromagnetic fields (e.g. X-rays)?

☐ No, never ☐ Sometimes

In your work, have you had regular exposure to cytostatics?

☐ No, never ☐ Sometimes

If you have checked the option "**No, never**" go to question **HAB01**. If you have checked "**Sometimes**", indicate the approximate number of cumulative months you have been exposed: ______months (*electromagnetic fields*) ______months (*cytostatics*)

LIFESTYLE

Height: ____ cm. Weight: ____ kg

How do you consider the physical activity you develop in your work?

☐ Light ☐ Moderate ☐ Hard ☐ Very hard

How many hours did you spend last week doing physical exercise in your free time?

_____ hours doing physical exercise the last week.

FAMILY RESPONSIBILITY

Do you have children under the age of 14? ☐ Yes ☐ No How many?: ___

Do you have dependents family (elderly, disabled or family members with a serious illness) in your care? ☐ Yes ☐ No How many?: ______

SLEEP AND REST

Do you have a regular sleep schedule? ☐ Yes ☐ No

Do you use any medication to get to sleep? ☐ Yes ☐ No ☐ No, but previously yes

TOBACCO EXPOSURE

Did you ever smoke cigarettes? ☐ Yes ☐ No

Do you currently smoke cigarettes?

☐ Yes, everyday ☐ Yes, some days ☐ No, I don’t smoke

How many cigarettes a day? (*Indicate 0 if you do not smoke*): ____ cigarettes

In your opinion, is the smoking ban complied in your job?

☐ Totally ☐ Almost always ☐ Hardly ever ☐ Never

How many hours a day are you exposed to tobacco smoke in your workplace? ___ hours

At work, how many hours a day do you spend with smokers? ____ hours

How often are you exposed to tobacco smoke inside your home?

☐Never or hardly ever ☐ Less than one hour a day

☐ Between 1 and 5 hours a day ☐ More than 5 hours a day

LABOUR DATA

CURRENT JOB

Indicate what type of corporation you are currently working on.

Type of system: ☐ Public system ☐ Private system

Attention level: ☐ Primary Care ☐ Specialized Care

Type of organization:

☐Public ☐ Public enterprise ☐ Consortium

☐Health alliance ☐ Private ☐ Other: ________________

How long have you been working at your current company?

☐ Less than 30 days. ☐ Between 1 and 6 months. ☐ Between 6 months and 2 years.

☐ 2 – 5 years. ☐ 5 – 10 years. ☐ More than 10 years.

Your current main job is: ☐ Full-time ☐ Part-time

Indicate the type of shift in the position you currently hold:

◻ Only morning	◻ Only afternoon/evening	◻ Only nights
◻ Morning and night	◻ Morning and afternoon	◻ Afternoon and nights
◻ Morning, afternoon and night	◻ 24h-shift	◻ 12h-shift (morning and night)
◻ Only morning + Eventual 24h	◻ Irregular	◻ Other:

PROFESSIONAL CAREER

Indicate the number of accumulated years you've been working on (throughout your life):

___ years

Indicate how many years you have worked regularly more than 3 nights per month:

___ years

Indicate the number of working nights accumulated throughout your life (approx.):

___ nights

Indicate the age at which you started performing night shifts (*indicate 0 if you have not performed night shifts*): ____ years

MEDICAL/SICK LEAVES

With regard to your sick leaves from work (in the last 12 months): *You can check more than one option.*

☐ Aproximately, I have been ___ days off due to illness in the last year.

☐ I haven't been on sick leave in the last year.

☐ I have had ___ sick leaves the last year.

☐ I've taken other types of sick leave (indicate type and number)_________________________________

If you have been on sick leave, please indicate the disease ______________________

SUGGESTIONS

If you have any suggestions, recommendations, consultations or would like to reflect any issues in the course of the questionnaire, please do so below:

THANK YOU VERY MUCH FOR YOUR INVALUABLE COLLABORATION.
